# Bird Radar Validation in the Field by Time-Referencing Line-Transect Surveys

**DOI:** 10.1371/journal.pone.0074129

**Published:** 2013-09-16

**Authors:** Adriaan M. Dokter, Martin J. Baptist, Bruno J. Ens, Karen L. Krijgsveld, E. Emiel van Loon

**Affiliations:** 1 Institute for Biodiversity and Ecosystem Dynamics, Computational Geo-Ecology, University of Amsterdam, Amsterdam, The Netherlands; 2 IMARES Wageningen UR, Den Hoorn, Texel, The Netherlands; 3 SOVON Dutch Centre for Field Ornithology, Coastal Ecology Team, Den Burg, Texel, The Netherlands; 4 Bureau Waardenburg, Culemborg, The Netherlands; University of Durham, United Kingdom

## Abstract

Track-while-scan bird radars are widely used in ornithological studies, but often the precise detection capabilities of these systems are unknown. Quantification of radar performance is essential to avoid observational biases, which requires practical methods for validating a radar’s detection capability in specific field settings. In this study a method to quantify the detection capability of a bird radar is presented, as well a demonstration of this method in a case study. By time-referencing line-transect surveys, visually identified birds were automatically linked to individual tracks using their transect crossing time. Detection probabilities were determined as the fraction of the total set of visual observations that could be linked to radar tracks. To avoid ambiguities in assigning radar tracks to visual observations, the observer’s accuracy in determining a bird’s transect crossing time was taken into account. The accuracy was determined by examining the effect of a time lag applied to the visual observations on the number of matches found with radar tracks. Effects of flight altitude, distance, surface substrate and species size on the detection probability by the radar were quantified in a marine intertidal study area. Detection probability varied strongly with all these factors, as well as species-specific flight behaviour. The effective detection range for single birds flying at low altitude for an X-band marine radar based system was estimated at ∼1.5 km. Within this range the fraction of individual flying birds that were detected by the radar was 0.50±0.06 with a detection bias towards higher flight altitudes, larger birds and high tide situations. Besides radar validation, which we consider essential when quantification of bird numbers is important, our method of linking radar tracks to ground-truthed field observations can facilitate species-specific studies using surveillance radars. The methodology may prove equally useful for optimising tracking algorithms.

## Introduction

While radar techniques have played a central role in the study of free flying birds ever since the technique was first applied in ornithology [1], [2], only recently the information technology has become established that allows storage and automated processing of the very large data flows generated by radars. This has sparked new types of ornithological radar studies, characterised by the possibilities of quantitative analysis based on large data sets in combination with predictive statistical modelling, e.g. [3]–[11]. With the commercial development of several off-the-shelve systems based on marine radars, bird radars have come available to a wide public of ecologists and conservationists [12]–[16]. The applied use of radar has ever increased, through the raised concern about the impact on bird populations of collision mortality with man-made structures such as wind farms and power lines [5], [11], [14], [17], [18], as well as to mitigate bird collision risks in aviation, which have increased dramatically during the last few decades [19], [20].

A major hurdle for quantitative studies is that often the detection capabilities of bird radars are poorly known [21], [22]. Many systems can be considered ‘black boxes’ of which the detection capabilities and limitations are poorly specified, making interpretation of the output in terms of animal targets difficult and prone to observational biases. Furthermore, the performance of a radar is dependent on a multitude of factors, such as the type of birds studied, their flight behaviour, the terrain of the study site and meteorological condition [21]–[25]. This underscores the need for practical methods for validating a radar’s detection capability in specific field settings, which is the topic of this paper.

Our validation approach consists of determining which fraction of a set of ground-truthed field observations, as a function of bird characteristics like species, distance, flight altitude etc., can be related to radar targets. Links between radar tracks and visual observations have been made manually in many radar studies, either by tracking radars with mounted parallel telescopes, or by radar operators pointing out tracks to nearby visual observers [2], [7], [21], [22], [26]. However, as soon as visual observers are positioned at certain distance from the radar and/or bird movements are numerous, it quickly becomes impossible to manually link visual observations to their respective radar targets.

To be able to link radar targets, the position of free flying birds needs to be determined in the field, such that at a later stage it can be verified whether a radar track was recorded at that same position and moment in time. Although determining the position of animals in the field is generally difficult and prone to estimation errors [27]–[29], the moment of crossing a line transect is one of the few types of positional information that can be quantified routinely and accurately. Line transects can be easily defined in the field by observers looking towards fixed visual landmarks near the horizon, such as towers, trees, buoys or wind turbines. The instant at which a bird crosses such a line transect is well-defined, which we will refer to as the visually determined transect crossing time 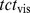. Field observers may record these instants relatively accurately using a GPS-referenced clock for all birds passing the transect, thereby building a ground-truthed set of partially geolocated observations.

We direct our method primarily towards validation of surveillance radars operating in track-while-scan mode, the standard operation of most portable marine radars and air traffic control radars. The validation is designed for field situations in which visual observers can monitor transects with a view of various flight altitudes, sufficient to monitor the main flux of birds over a certain range of distances and altitudes. As long as birds pass the transect one by one, that is outside periods of extremely numerous movements, a visual observations and its corresponding radar track will share the same transect crossing time, by which the two can be linked.

We will use ‘distance’ to denote the distance of a bird to an observer, and ‘range’ as the distance of a bird to the position of the radar throughout.

## Materials and Methods

### Ethics Statement

Permission for accessing the tidal flats of the Balgzand study area was issued by the Provincie Noord-Holland. Permission for accessing all other count sites was issued by the Royal Netherlands Navy.

### Bird Radar

We used a prototype track-while-scan bird radar provided by Robin Radar Systems, which was based on an X-band Furuno marine radar (magnetron-amplified radiation, 25 kW power output, 8 feet horizontally scanning T-bar antenna). The nominal beam width was 1 degree in the horizontal dimension, versus 20 degrees in the vertical dimension. The radar processing uses adaptive ground clutter filtering through subtracting from the raw reflectivity data a land clutter mask, which is continuously updated by averaging in a proportion of 0.1 of the last acquired reflectivity image. The subtraction of background clutter improves tracking of birds on top of ground clutter signals. Radar tracks are automatically identified by a tracker algorithm and stored in an SQL database. The system can be considered state of the art, in the sense that it is fully automatic and uses dedicated clutter suppression techniques optimised for the detection of birds. A detailed description of tracker and clutter suppression algorithms is beyond the scope of this paper, and is partly proprietary information of Robin Radar Systems.

Radar tracks had a minimum track time of 5 seconds. For each track the air speed was calculated by subtracting the wind speed vector. We accepted tracks with air speeds up to 25 m s^−1^, which is above the maximum air speed of most species in our study area [30]. We assume the threshold is sufficiently high to tolerate some potential deviations with the true airspeed due to altitudinal changes in wind. The threshold was applied to discard tracks of frequently passing helicopters, as well as to reduce the number of tracks related to sea clutter at short range, which often showed unrealistically high air speeds.

### Time-referenced Line-transect Surveys

**Figure 1:**
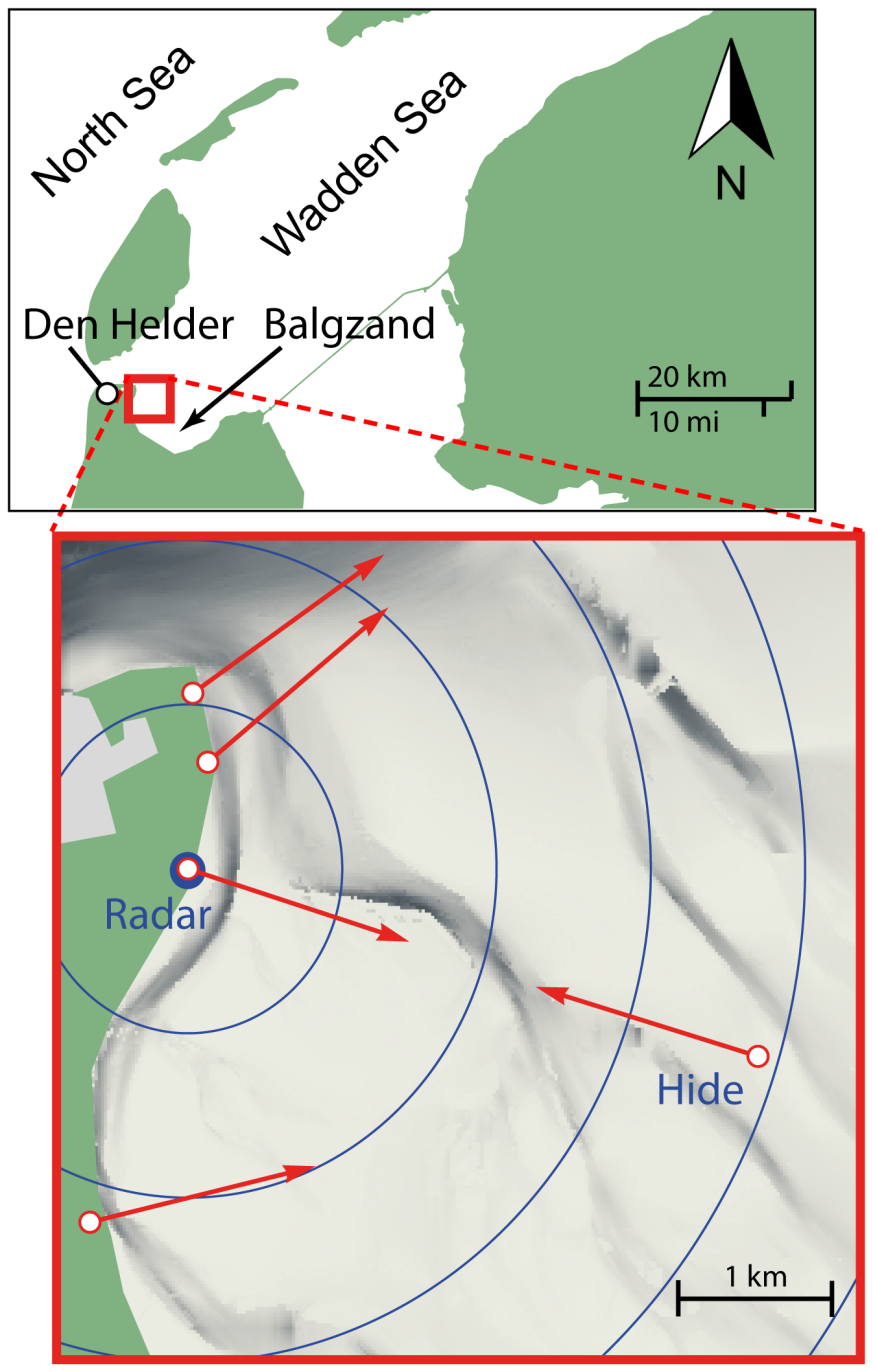
Study area and position of the bird radar. Map of the north-western part of the Balgzand intertidal area in the Wadden Sea, the Netherlands, showing the position of the radar (blue dot) and the transects used by visual observers (red arrows into the direction of observation). Concentric circles around the radar position are separated by a range of 1 km.

Field observations took place in 2010 on 17-Mar 9∶00–18∶00 (UTC), 18-Mar 6∶30–17∶00, 19-Mar 6∶30–14∶30, 10-May 7∶30–16∶30, 11-May 5∶00–15∶30, 31-Aug 8∶00–16∶00, 1-Sep 7∶00–14∶30, 2-Sep 6∶00–13∶30 on 3–4 transects simultaneously. Monitored transects are indicated in Fig. 1 by red arrows. Transects were monitored by observer pairs on the ground, except for the transect starting on the tidal flats, which was monitored from a hide at 4 m above ground.

The survey protocol was designed as follows. One observer monitored the transect and one field assistant wrote down the observations. Observers used standard binoculars of 10× magnification. For each bird crossing the transect, the field observer called out the species name to the field assistant, who wrote down the transect crossing time from the clock of a hand-held GPS device. Counts were interrupted when bird movements were too numerous to maintain protocol. In addition, the observer recorded whether the transect was crossed either from the left or from the right, and provided an estimate of the bird’s flight altitude and distance, according to the categories listed in Table 1. Proper assignment to a distance class was aided by defining the transitions between classes in terms of (natural) landmarks, or by choosing a transect perpendicular to the line of sight of the radar, which guarantees that all observations at that transect are made at approximately the same radar distance (most southerly transect). For dense groups of birds, a single transect crossing time was recorded together with the flock size. Since the large majority of observations related to individually flying birds, our analysis will focus on single birds only. Each transect was actively surveyed for 10 minutes every half hour, making up for a total active observation time of 25 hours (∼6 hours/transect). Observers were randomised over the transects between days.

**Table 1:** Altitude and distance classes used in the line transect surveys.

Distance to observer [km]				Flight altitude [m]			
class	range	n	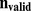	class	range	n	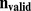
1	0–0.2	1033	418	1	0–2	2629	840
2	0.2–0.5	2545	867	2	2–20	5131	1602
3	0.5–1.0	3255	942	3	20–100	1103	422
4	1.0–1.5	1521	478	4	100–500	80	32
5	1.5–3.0	614	202				

Distance and altitude ranges defining the distance and altitude classes. In addition, 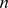 indicates the total number of observations per class, and 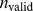 the number of valid (sufficiently time-separated) observations used for the validation.

Consecutive observations are labelled by index 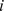 and we will refer to the corresponding transect crossing time as 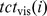. Observer teams can determine a bird’s transect crossing only up to a finite accuracy. Therefore 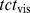 will be a random variable, for which the residuals with the true transect crossing time will be assumed to follow a normal distribution 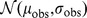, with an observer’s standard error 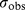 and a potential mean time-delay that observers require to write down the transect crossing time 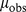.

The full set of visual observations we refer to as 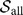. For validation purposes we will only consider observations which are well time-separated from the preceding and subsequent observations along the same transect, by requiring a minimum time separation 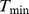 of consecutive observations:
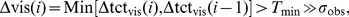
(1)

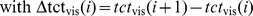



The subset of visual observations for which Eq. 1 holds (for certain choice of 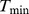) we will refer to as 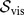. For this set the index i is re-indexed such that it denotes consecutive observations out of the full set of visual observations 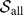 for which 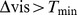.

### Linking Radar and Visual Observations

Given a visual observation 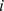 of a bird crossing a transect at certain time 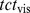 and a radar track 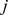 crossing the same transect at time 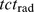, the time difference between these transect crossing times equals
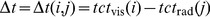
(2)

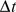
 is assumed to follow a normal distribution 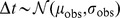.

The link algorithm for assigning visual observations to radar tracks is set up as follows. For each observation 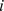 with assigned distance class 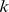 (see Table 1), we select candidate tracks 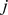 which satisfy 3 requirements:

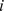
 and 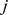 intersect the transect into the same direction
2. the transect crossing of track 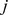 occurs at an observer distance between 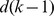 and 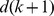, where 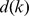 equals the central observer distance of the distance range in class 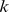. This requirement selects only weakly on the estimated distance of a bird by the field observer, since we expect distance estimation through visual observation to be prone to estimation errors.

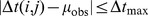
, i.e. the transect crossing time of radar and visual observation should be equal, within a tolerance 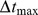.

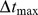
 should not be larger than 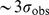, as this will unnecessarily increase the possibility of mismatches. If 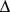
t_max_≲3σ_obs_, some matches will not be found, for which needs to be corrected when calculating probabilities of detection. We may correct for this reduction by realising that the fraction of found matches, 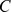, equals.
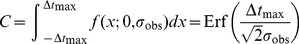
(3)where 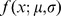 is the probability density function of a normal distribution with mean 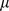 and standard deviation 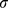. The true number of matches is thus found by dividing the detected number of matches by 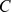.

The combined set of candidate tracks for all observations in 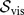 we will call 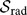. This set potentially includes multiple tracks as candidate match for the same visual observation, or single tracks as candidate match for multiple visual observations (though by our requirement of properly time-separated subsequent visual observations and small 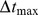 this occurs rarely in practice). From the set 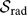 we construct a final subset of track - visual observation pairs 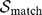 containing valid links between visual observations and radar tracks: we select without replacement the set of pairs 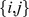 (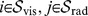 ), that minimises 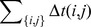, where the sum runs over all pairs in set 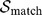. Visual observations left unpaired add the maximum penalty of 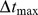 to the sum.

### Determining the Observer Timing Accuracy

To determine the observer timing precision and accuracy, i.e. the magnitude of respectively 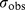 and 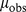, we evaluate the effect of an imposed time lag 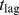 between visual observations and the set of radar tracks, by transforming all 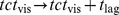. We run the link algorithm on the full set of visual observations 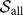 (not 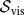) and calculate how the number of matches found depends on 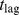. We will refer to this response as the ‘lag-curve’. The lag curve will show a maximum at 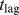 = 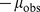, since then the visual and radar observations are optimally aligned in time. When 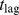 is increased, visual and radar tracks will become misaligned in time, and the number of found matches will decrease with a rate that depends on the magnitude of 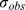.

Formally, the shape of the lag curve depends both on the observer timing accuracy, assumed to follow a normal probability distribution 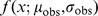, and the requirement 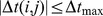 for candidate matches (requirement 3 previous section). This requirement is equivalent to assigning probabilities to radar tracks for potential linking according to the following block curve:
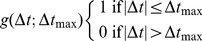
(4)


The joint probability function for observer timing errors and radar track matching errors is calculated by a convolution between the two separate probability functions:
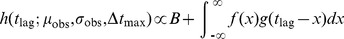
(5)

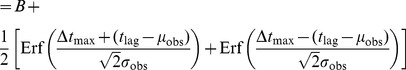
with 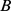 a baseline level of matches found in conditions of full time-misalignment. We find 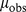 and 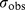 by fitting the observed lag curve to Eq. 5 using a least-squares criterium. For the width of the lag curve to be dominated by 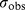 and not by 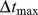, we run the link algorithm with 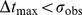, in our case 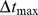 = 2 s. When required, more than one lag curve may be calculated, e.g. for specific observers and altitude and distance categories.

### Study Area and Environmental Data

The radar was stationed at the naval base of Den Helder, 52.9534°N, 4.8013°E, neighbouring the Balgzand protected intertidal area, the most south-western part of the Wadden Sea, as illustrated in Fig. 1. The mudflats of the Balgzand are alternatingly flooded and exposed under influence of the tides. Tidal height was measured by the tidal station of Den Helder (52.9644°N, 4.74499°E). A bathymetric map of this area (20 m resolution) was provided by Rijkswaterstaat, Ministry of Infrastructure and the Environment (Vaklodingen 2003–2008). A distance class of a transect was considered flooded when the tidal height exceeded the bathymetric height for at least 50% of the sector, and was otherwise considered exposed. Wind speed and direction at 10 m above ground level were obtained from nearby meteorological station De Kooy (52.93 N, 4.78 E) operated by the Dutch Meteorological Institute (KNMI). Bird air speeds were calculated by subtracting the wind velocity vector from the radar track velocity vector, calculated as an average over all segments of the radar track.

### Statistical Modeling

We constructed logistic generalised additive models using the gam function of the mgcv package for the R language of statistical computing [31], [32], using thin plate regression splines as smooth terms [33]. We tested models for the categorial probability of detection (POD) (0/1 for a undetected/detected visual observation) in terms of up to 5 dependent variables: range (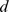), flight altitude (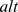), body mass (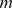), species (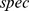) and surface substrate state (flooded or emerged) (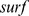). Sex-averaged mean body masses for each species were taken from Dunning [34]. We took as the range 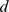 of a visual observation at a certain transect the mean range of its distance class. Model performance was assessed in terms of AIC values [31], [35]. We calculated binomial proportion confidence intervals using the Wilson score interval, at a confidence level of 95%.

## Results

**Figure 2:**
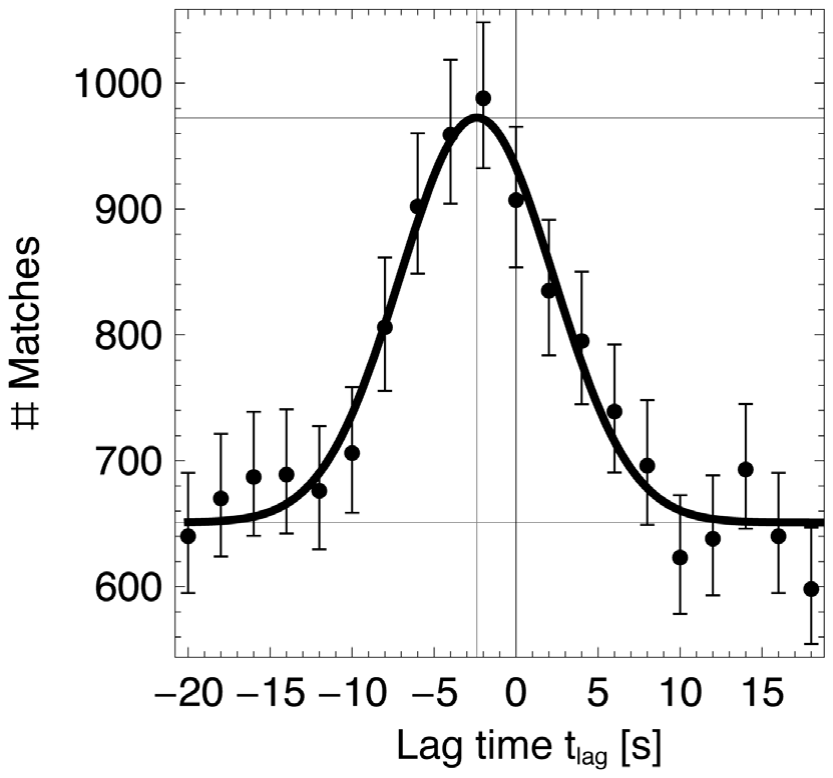
Lag curve. Number of radar tracks that could be matched to a visual observation, as a function of an imposed time lag 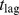 between visual observations and the set of radar tracks. This lag curve was calculated for the full visual observation set 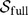. The solid line is a fit to Eq. 5, giving 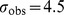 s and 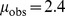 s.

To determine the observer’s timing accuracy we calculated the lag curve for the full set of field observations 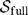, as illustrated in Fig. 2. The solid line indicates a least-squares fit using Eq. 5. This fit quantified the parameters for the observer timing accuracy at values 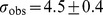 s and 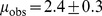 s. Observers thus reported a bird’s transect crossing with an average delay of 2.4 s and with a standard deviation of 4.5 s. By calculating and comparing separate lag curves for nearby (≤500 m, distance classes 1–2) and distant (> 500 m, distance classes 3–5) flying birds, we verified that the ability of observers to time a transect crossing did not vary significantly with distance. For nearby flying birds we found 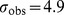 s and 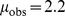 s and for distant flying birds we found 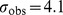 s and 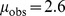 s. Neither the observer timing accuracies 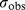 are significantly different (Two-sided F-test, F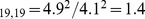, p = 0.4), nor the delay 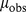 (Two-sided t-test, t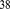 = 0.2, p = 0.8). Because the values 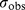 and 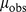 did not vary significantly with distance class or observer pair, they were kept constant throughout the validation (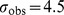 s and 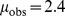 s).

**Figure 3:**
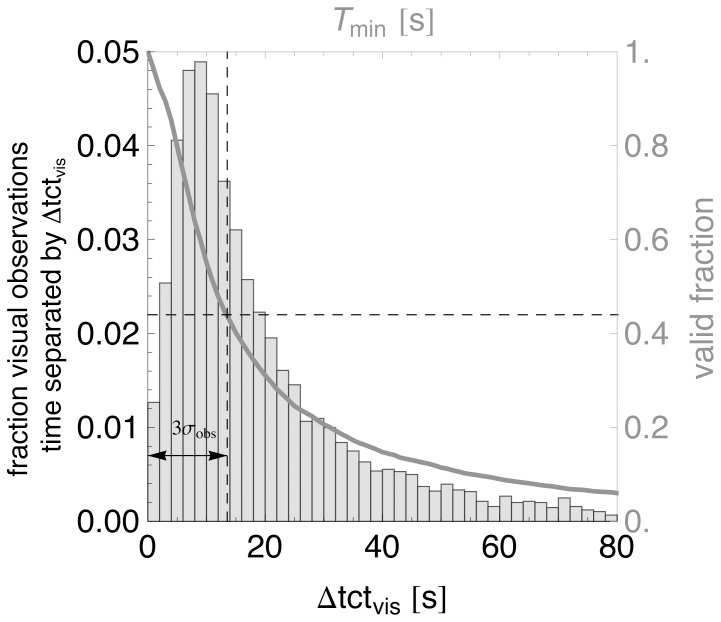
Time difference between consecutive visual observations. Probability density histogram (2 s bins) for the time difference between consecutive observed transect crossings (bottom/left axes). The gray curve shows the fraction of valid observations for different values of 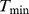, i.e. the fraction observations for which both the following and preceding observation are found at a time interval larger than 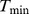 (top/right axes). The dashed vertical line indicates 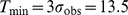 s, as used in this study. This value implies that a fraction of 0.43 of the total number of observations is used for the validation (dashed horizontal line).

We subsequently selected a set of observations 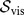 to be used for validation that were well time-separated, as set by the parameter 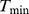. The time separation of the observations is illustrated in Fig. 3, which shows visually recorded transect crossings followed each other rapidly with a most frequent time spacing of 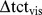 = 8 s. The grey line illustrates the fraction of observations that are available for validation as a function of the minimum time spacing 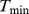 (i.e. observations satisfying Eq. 1, with crossing towards left and right treated separately). As a compromise between sufficient time separation and a sufficiently large validation dataset 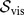 we chose 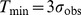 = 13.5 s.

The final parameter of the link algorithm to be set is 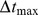, the maximum difference in transect crossing time for a valid link between a radar and visual observation pair. When 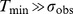, this parameter is preferably set at 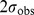, such that only 5% of the potential links will not be found and the correction factor C (Eq. 3) is small. In our case 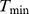 is only three times 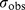 and we need to choose a smaller 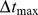. To make sure a radar track cannot be incorrectly linked to any preceding or subsequent visual observation to which it does not belong, we set 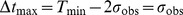, i.e. radar track and visual observation pairs can only be linked in a time-window not overlapping with the 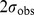 probability range of occurrence of any preceding or subsequent visual observation, which limits the possibility of mismatches. This fixes the correction factor at 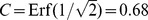. We finally run the link algorithm on the observational dataset 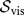, whose transect crossing times were time shifted by subtraction of 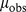 to correct for the average time delay between observing and writing down transect crossing times by field observers.

We applied logistic generalised additive modelling to assess the link algorithm output and to quantify the probability of detection (POD) of the radar system, in terms of various explanatory variables. The observed POD equals the proportion of birds seen crossing the transect by a field observer, that was also detected by the radar (as determined by the link algorithm with parameters derived from the lag curve as discussed above). Various tested models are summarised in Table 2. The POD following from the best GAM model can be written as follows:
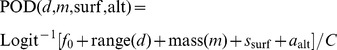
(6)with surf
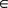
{dry,wet}, alt
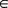
{1,2,3,4}, and range(d) and mass(m) two cubic regression smooth terms dependent on range 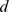 [km] and bird mass 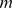 [g]. The inverse logit function equals 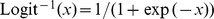. The smooth terms could be parametrised by a power series up to fifth order (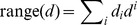 and 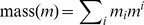, 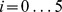) for bird masses up to 2 kg and ranges up to 4 km. All model parameters are reported in Table 3.

**Table 2:** GAM models for the probability of detection compared by AIC.

id	GAM formula (Logit link)	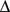 AIC	df	Deviance	
1	POD ∼ d+m+surf+alt	0	12	0	
2	POD ∼ d+m+surf:alt	4	15	2	
3	POD ∼ d+m:alt+surf	5	18	8	
4	POD ∼ d:alt+m+surf	8	20	9	
5	POD ∼ d+m+alt	9	11	−11	***
6	POD ∼ d+alt+surf	18	9	−24	***
7	POD ∼ d+spec+surf+alt	20	57	72	**
8	POD ∼ d+alt	26	8	−35	***
9	POD ∼ d+m+surf	28	9	−33	***
10	POD ∼ d+m	39	8	−47	***
11	POD ∼ d	58	6	−70	***

POD = probability of detection, d = distance, m = mass, surf = surface dry/wet, alt = altitude, spec = species. df gives the (estimated) degrees of freedom of the model. “:” indicates an interaction of all the variables and factors appearing in the term. The degrees of freedom for the mass smooth term was restricted to 5. Stars indicate the significance of a model comparison according to a Chi-squared test between each model and the best (first) model (under assumption of df),

*** p<0.001,

** p<0.01,

* p<0.05.

**Table 3:** Coefficients of the best GAM model for the probability of detection.

	Smooth terms		Parametric coefficients		
i	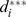	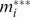	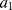	0	–
0	1.10569	−0.447822	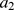	0.455609	***
1	1.31395	1.007610	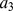	0.894142	***
2	−1.66196	0.347843	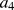	1.635100	**
3	0.18914	−0.930752	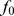	−1.990540	**
4	0.0773222	0.408188	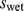	0	–
5	−0.014866	−0.0563531	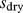	−0.491847	**

Stars indicate significance of each term according to a Wald test against the null hypothesis that the term is zero (*** p<0.001, ** p<0.01, * p<0.05).

**Figure 4:**
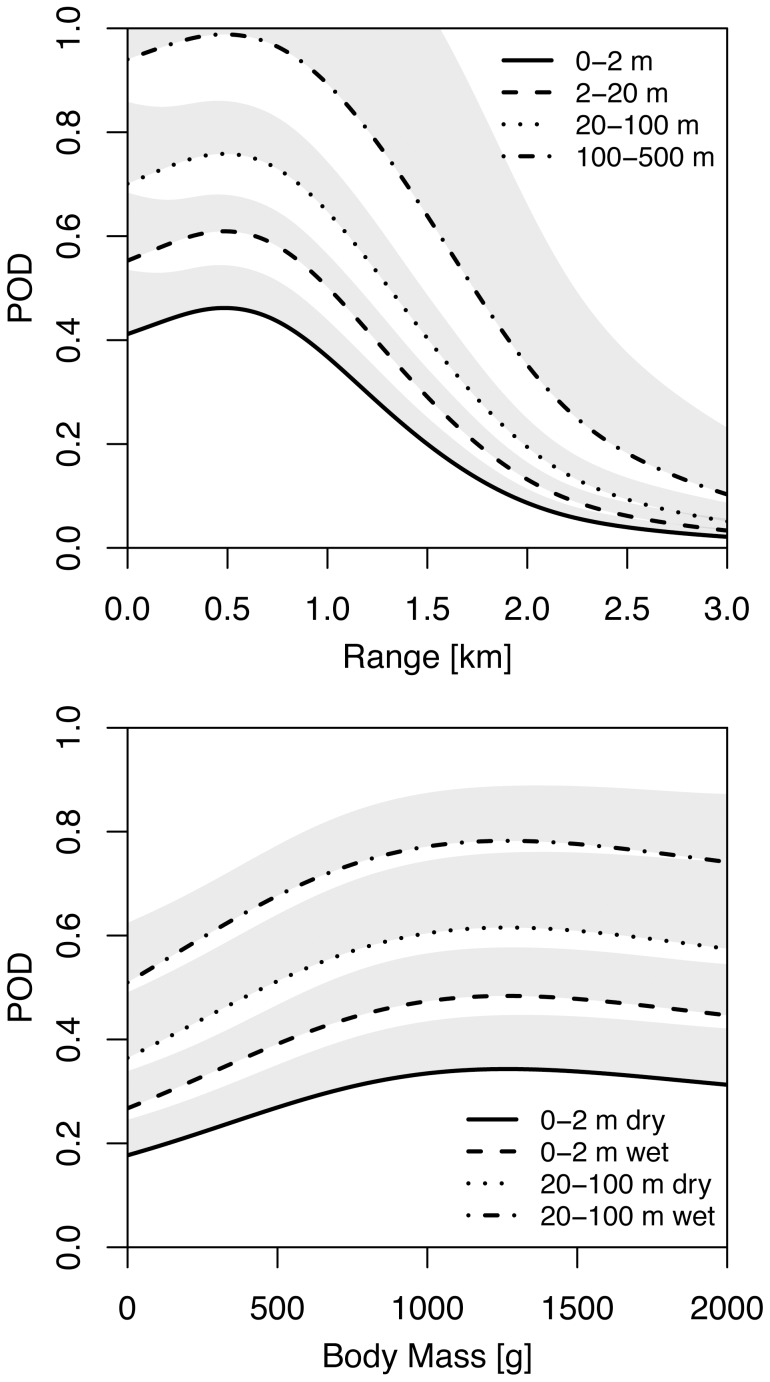
GAM predictions for the probability of detection (POD). Top: the effect of range for four altitude categories for the median size of birds in our study area (0.4 kg) above a flooded surface. Bottom: the effect of body mass for two altitude categories, both above a dry and wet surface. To illustrate the uncertainty estimates of POD, the area between the mean and the upper 1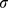 bound of the confidence interval is indicated in grey.

In Fig. 4 the modelled POD is plotted as a function of bird mass and range for various flight altitudes and ground surface states. Even at close ranges the detection probability stays below 1, except for the highest flight altitude category 100–500 m. Around 1.5 km range the POD drops to 50% of its peak value, which we will consider the approximate functional range of this radar. The POD also linearly increases with body mass up to masses of about 1 kg, after which the POD levels off to a near constant value. The effect of the surface substrate is considerable, with an increase in POD around 30% for flooded compared to exposed intertidal flat.

**Figure 5:**
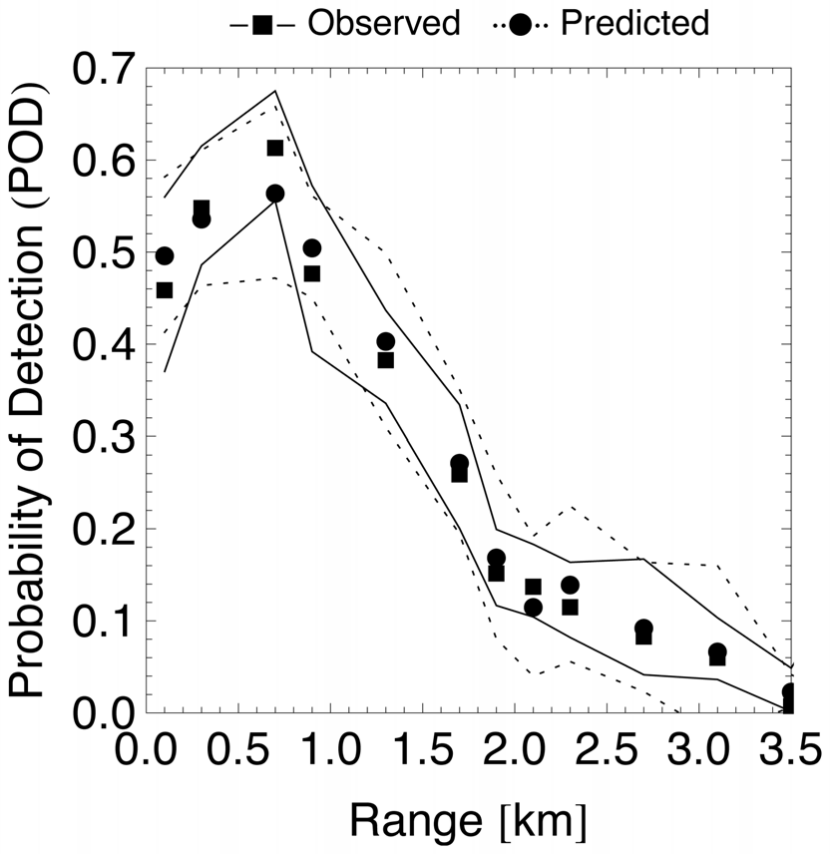
Average probability of detection (POD) as a function of range. Each scatter point refers to a distinct distance class of one of the transects and its corresponding subset of visual observations from 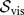, drawn on the horizontal axis at its mean range. The modelled POD equals the mean GAM prediction for these observations. The observed POD equals the proportion of these observations that could be matched to a radar track directly. Lines indicate the upper and lower 1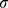 confidence intervals.

We may use the GAM model of Eq. 6 to predict a POD for all birds in 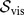. Filling out the parameters of each observation in the GAM, the model gives a POD and standard deviation per observation. From these values we calculated mean POD values for each distance class in each transect, which are plotted as the modelled POD in Fig. 5. The same figure shows the observed POD, which equals the proportion of observations that could be matched to a radar track for each distance class in each transect directly (in this case confidence intervals were calculated using the Wilson score interval). Taking into account only observations within the functional range of 1.5 km, we find that the radar tracks 50±6% of all bird movements.

**Figure 6:**
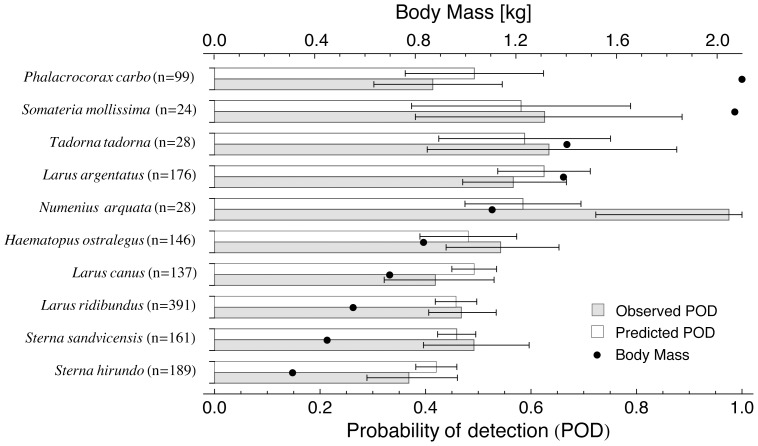
Average probability of detection (POD) per species in the radar range 0–1500 m. POD values are shown for the 10 most frequently observed species, from top to bottom ordered by body mass: Great Cormorant, Common Eider, Common Shelduck, European Herring Gull, Eurasian Curlew, Eurasian Oystercatcher, Common Gull, Black-headed Gull, Sandwich Tern and Common Tern. The modelled POD equals the mean GAM prediction for all visual observations within this 0–1500 m range. The observed POD equals the proportion of these observations that could be matched to a radar track directly. Black dots indicate the average body mass per species.

Fig. 6 shows the observed and predicted detected fraction for the 10 most commonly observed bird species, ordered from large to small species from top to bottom. The largest birds are not necessarily detected by the radar with the highest probability.

## Discussion

Using a validation approach based on time-referencing transect counts, we have obtained a probability of detection (POD) function for a track-while-scan bird radar in terms of bird size, flight altitude, range and surface substrate, at a specific field site. To our knowledge, such a POD function for bird targets has not been determined earlier for track-while-scan surveillance radars. The POD function is essential to quantify the limits and conditions where a radar can be operated without introducing observational biases, which is a prerequisite for quantitative studies [21]. Bias corrections based on the POD function can be applied where necessary to obtain a corrected count of the bird numbers aloft, such that studies no longer need to rely on unspecified indices for the intensity of bird movements, e.g. [36].

We will discuss in detail the most striking validation outcomes. First, the operational range of the radar for the detection of single birds is relatively small at 1.5 km. In many studies similar radars have been operated up too much longer ranges, e.g. [5], [24], [25], [36], [37], suggesting the radar observations in these studies may be biased towards higher flight altitudes and/or larger flocks than single birds. Repeating the validation procedure on different radar systems is however required to enable a true comparison of performance, which we strongly encourage.

We find the radar detection capability above a water surface is better than above a land surface, which in this particular study can be explained from a stronger clutter background from land surfaces compared to water surfaces. As long as a water surface is relatively smooth, as applies to the shallow sea in our study area, it acts as a reflector for radio waves, and reradiated energy from the surface is directed primarily away from the radar [38]. However, clutter from water surfaces may become severe when the water surface roughness increases, e.g. at open sea in conditions with high waves and strong wind, when the detection probability may become lower than above stable land surfaces [24], [25]. We did not investigate effects of sea state on the probability of detection, but the applied regression techniques do allow testing of such factors.

Despite the adaptive clutter filtering applied in the radar processing, we find that flight altitude was a dominant factor determining a bird’s probability of detection. For flight altitudes near the surface the detection probability remains below 1 at all ranges and for all species of birds. This effect points to an increased difficulty to distinguish a bird from the background of ground clutter signals the closer it flies to the surface, a well-known limitation of bird radar systems, e.g. see [22], [23]. Due to correlations between species and specific flight altitudes and surface substrates, larger bird species are not necessarily detected more frequently than smaller bird species, as illustrated in Fig. 6. For example, the Great Cormorant (*Phalacrocorax carbo*) is the largest bird frequently observed in our study area, but this species has the habit of skimming low over the sea surface, making its detection probability similar to that of the Common Tern (*Sterna hirundo*), the smallest regularly observed species. The relatively high detection probability for Eurasian Curlew (*Numenius arquata*) may lie in the fact this species was observed to fly relatively high, but also has a highly directed and fast flight. Similar-sized gulls, showing similar flight altitudes but lower POD, may have the tendency to show more erratic flight behaviour while foraging, having a potential negative impact on the probability of detection. Flight behaviour may thus be an important additional factor determining the POD.

A validation design based on matching transect crossing times between visual observations and radar tracks has the important advantage that the validation outcome will not depend critically on the detection and distance estimation capabilities of field observers, for several reasons. First, transect crossing times can be accurately estimated, also at larger distances, which we concluded from similarly shaped lag curves for close and distant visual observations. Second, the validation is based only on positive detections by field observers. Therefore it is not required to continually record all birds while monitoring a transect, which can be hard in practice when movements are numerous or when the observer distance is large. Third, although distance estimation is used to assign the visual observation to one of the distance classes, this information is explicitly not used in linking the observation to its corresponding radar track. Hereby we allow for a certain degree of error in the distance estimation by observers, and exclude the possibility that a properly detected radar track is not linked to its corresponding visual observations because of a poor distance estimate.

We would like to emphasise that the presented method for linking radar tracks and visual observations is intended primarily for study sites where birds fly low within visual range of ground observers. Many bird movements occur at low altitude, especially during short-distance foraging trips, and the low altitude regime has a high practical relevance (e.g. for mitigating bird collisions with turbines and aircraft). Further limitations of the method are related to the capabilities of field observers to correctly categorise the different distance and altitude classes, which may be difficult in the absence of visual landmarks [28], but is achievable by experienced observers [29].

For multiple reasons it is recommended to achieve a high observer accuracy, i.e. a 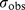 as small as possible. First, a high observer accuracy permits using more closely time-separated observations for validation (i.e. permits a smaller choice of 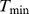 such that Eq. 1 holds for more observations). In our case 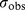 was relatively large at 4.5 s, which resulted in a high fraction of discarded observations (see Fig. 3), especially during periods with very numerous bird movements. Second, the use of closely time-separated observations allows inclusion of events with very high traffic rates in the validation. This may permit a quantification of how the detection function of the radar varies in relation to bird traffic density itself. A decrease in detection probability may occur at very high traffic rates, when the spatial resolution of the radar becomes insufficient to resolve all targets individually, and the radar tracker may start merging several birds or flocks into single objects. Third, a small 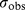 permits a small time window around a visual observation in which to search for its corresponding radar track (i.e. a small 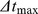). This limits the possibility of potential mismatches to birds that were accidentally missed by field observers. We therefore recommend the use of digital event recorders operated by the visual observer to time the transect crossing times as accurately as possible, such that a smaller 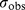 than reported in this study may be achieved.

While presented here primarily in the context of radar performance validation, time-referenced transect counts combined with a track linking algorithm have a broader applicability. First, the method may be used to optimise tracking algorithms. Through storing the raw radar signal during the validation and reprocessing the track extraction with different parameters or tracking algorithms, the link algorithm can be run repeatedly on the same visual observation data set, allowing comparison of validation results for different tracker settings. Second, the method may be used to study the distance estimation capabilities of field observers [29], whose accuracy is highly relevant for survey techniques such as distance sampling [27]. Finally, time-referencing transect counts may be used as a general strategy for routinely linking large numbers of radar tracks to their respective species identity, thereby allowing species-specific studies using surveillance radars.
